# Bisquinolinium compounds induce quadruplex-specific transcriptome changes in HeLa S3 cell lines

**DOI:** 10.1186/1756-0500-5-138

**Published:** 2012-03-13

**Authors:** Rashi Halder, Jean-Francois Riou, Marie-Paule Teulade-Fichou, Tancred Frickey, Jörg S Hartig

**Affiliations:** 1 Department of Chemistry and Konstanz Research School Chemical Biology (KoRS-CB), University of Konstanz, Universitätsstraße 10, 78457 Konstanz, Germany; 2 Structure des Acides Nucléiques, Télomères et Evolution, INSERM U565, CNRS UMR 7196, Muséum National d'Histoire Naturelle, 43 rue Cuvier, 75231 Paris cedex 05, France; 3 Institut Curie, UMR 176-CNRS, Bât 110, Université Paris-Sud, 91405 Orsay, France; 4 Department of Biology, University of Konstanz, Universitätsstraße 10, 78457 Konstanz, Germany

## Abstract

**Background:**

Guanosine rich sequences capable of forming G-quadruplex (G4) motifs are enriched near the gene transcription start site (TSS) in the human genome. When probed at the single gene level, G-quadruplex motifs residing in promoter regions show substantial effects on gene transcription. Moreover, further changes in transcription levels are noticed when G4-motifs are targeted with G-quadruplex-specific small molecules.

**Results:**

Global studies concerning general changes of the transcriptome via targeting promoter-based G-quadruplex motifs are very limited and have so far only been carried out with compounds displaying weak selectivity for quadruplex sequences. Here we utilize two G-quadruplex-specific bisquinolinium derivatives PhenDC3 and 360A and investigate their effects on the expression of the HeLa S3 transcriptome. Our results show wide-spread changes in the transcriptome with specificity for genes with G-quadruplex motifs near their transcription start sites (TSS). Using real-time PCR we further confirmed the specificity of PhenDC3 and 360A as potent molecules to target G-quadruplex-regulated genes.

**Conclusions:**

Specific effects on quadruplex-containing genes have been observed utilizing whole-transcriptome analysis upon treatment of cultured cells with quadruplex-selective bisquinolinium compounds.

## Background

G-quadruplex motifs are four-stranded DNA conformations adopted by certain guanosine-rich sequences [1] abundant in the human genome [2,3]. Interestingly, these G-quadruplex motif-forming sequences show a pronounced positional bias and consequently a wide range of biological functions have been predicted for G-quadruplex motifs, including roles in transcription [4-6], translation [7,8], replication [9], nucleosome positioning [10,11], CpG methylation [12], recombination [13] and splicing [14]. Due to their high over-representation near transcription start sites (TSS) [15], the role of G-quadruplex motifs as transcriptional regulators has been studied most intensively (reviewed in [16-19]). The first experimentally verified biological function for a G-quadruplex motif was the transcriptional regulation of the *c-MYC *gene [20,21]. Similar modulatory effects in transcription have since been demonstrated for quadruplex motifs in the promoter regions of *c-MYB *[22], *VEGF *[23], *c-KIT *[24], *KRAS *[25], *HRAS *[26], *PDGFR-β *[27] and *BCL-2 *[28] expression. In several studies a small molecule specific to G-quadruplex motifs was used to demonstrate that quadruplex formation *in vivo *results in a perturbation of transcription (reviewed in [19,29,30]). However, since more than 55% of the genes in the human genome harbor at least one potential G-quadruplex motif 1 kb up- or downstream of the TSS [5], it is of interest to investigate whether addition of quadruplex-interacting compounds affects transcription of many or most of these genes.

Using the G-quadruplex-stabilizing molecule TMPyP4 [31], Verma *et. al*. [5] and Mikami-Terao *et. al*. [32] showed genome-wide effects on transcription in HeLa S3 and K562 cells, respectively. However, the specificity of the observed effects can be questioned because TMPyP4 is reported to show only poor selectivity for G-quadruplex motifs compared to e.g. duplex DNA [33]. The two studies identified a rather small set of affected genes at prolonged (48 h) treatment using high (100 μM) concentrations of TMPyP4. In these studies, only 69 [5] and 87 [32] perturbations of mRNA levels were observed. Moreover, porphyrin molecules were reported to have cytotoxic effects starting at 50 μM [34,35] and are photoactive [36,37], potentially resulting in harmful side-effects. More recently, using a single-chain antibody specific to G-quadruplex motifs, Fernando *et. al*. [38] determined 1,767 significant differentially expressed genes in a HGC-27 cell line. It should be noted that the choice of the cell line has very likely a strong impact on the outcome of whole-transcriptome examinations as described here. For example, a direct comparison of differentially expressed genes upon compound incubation between two cell lines in our opinion makes only sense if the compared genes are indeed expressed in both cell lines. Hence the immediate comparison of transcripome datasets between different cell lines will result in many apparent contradictions that might result from different expression states of the genes in different cell lines. In a further study, G-quadruplex motifs were selectively pulled down from HT1080 cells using a biotinylated bisquinolinium derivative [39]. However, in the latter study, an assignment of preferential genomic location for G4-motifs was not achieved, thereby limiting the insights gained regarding the *in vivo*-function of G-quadruplex formation. In conclusion, further genome-wide studies of transcriptional changes mediated by quadruplex-specific compounds are needed in order to judge the extent to which G-quadruplexes influence transcription.

**Figure 1:**
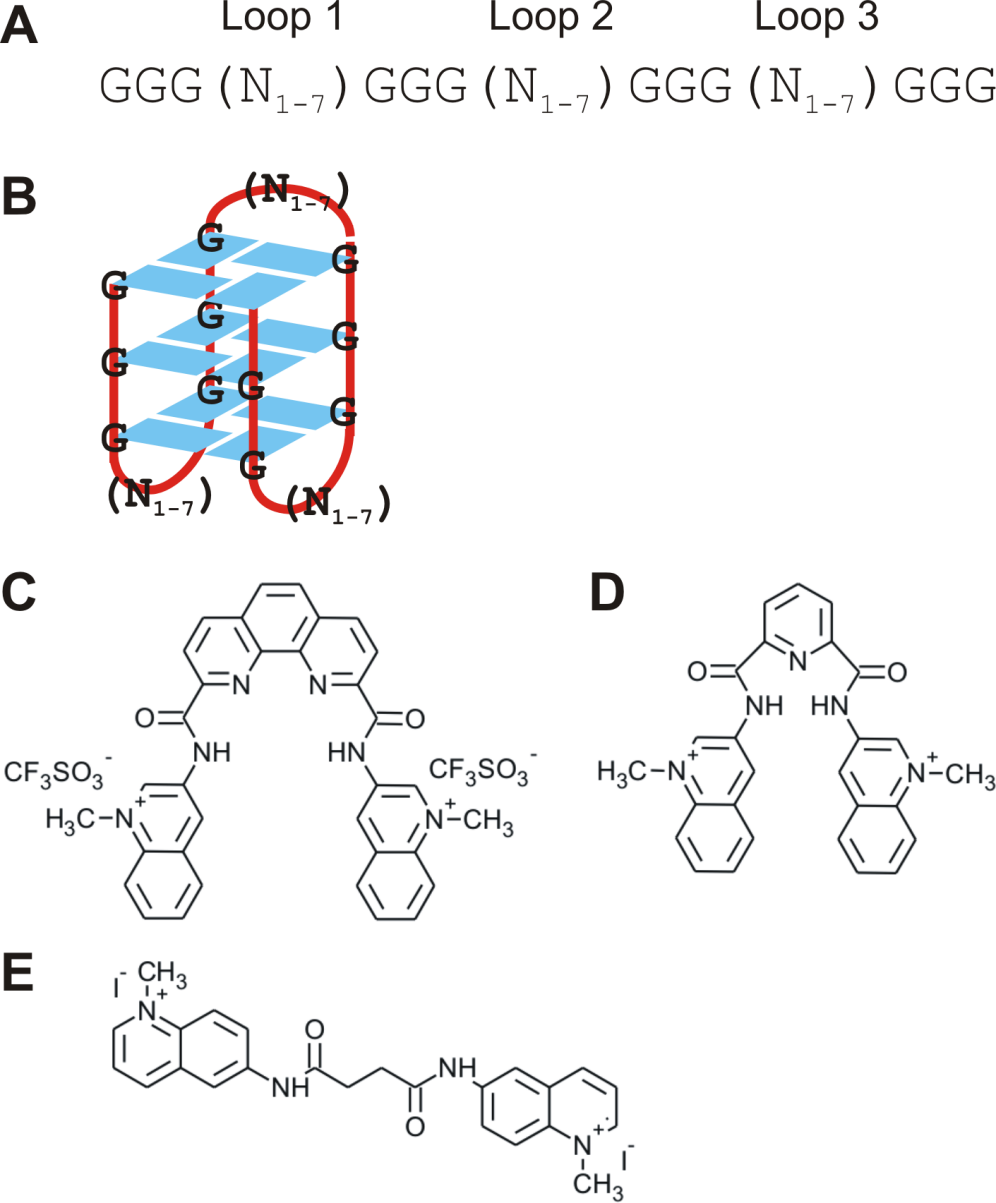
**Quadruplex sequences and bisquinolinium compounds:** A) Potential Quadruplex-forming sequence containing four G-tracts and variable loops 1-3. B) A possible quadruplex structure. C) PhenDC3. D) 360A. E) 8979A used in the study.

In the present work we investigate genome-wide effects on transcription in a HeLa S3 cell line via stabilizing G-quadruplex motifs using the quadruplex-selective bisquinolinium compounds PhenDC3 and 360A (Figure 1A-B). Both molecules derived from the triazine derivative 12459 [40] and have been extensively studied for their G-quadruplex specificity both *in vitro *[41-43] and *in vivo *[44-47]. In addition we used the bisquinolinium compound 8979A as a control as this compound also contains two quinolinium residues (see Figure 1C) but is known for its poor affinity and selectivity for DNA G-quadruplex motifs [43]. Of the 24,651 genes probed in this work by a transcriptome array, 2,212 and 526 genes significantly changed their expression when treated with PhenDC3 or 360A, respectively. Of these, 81.5% and 70% had at least one G-quadruplex motif near their TSS. We confirmed the specificity of the target G-quadruplexes by real-time PCR for 8 individual genes.

## Results and discussion

### Transcriptome profiling of HeLa S3 cells treated with PhenDC3 or 360A

**Figure 2:**
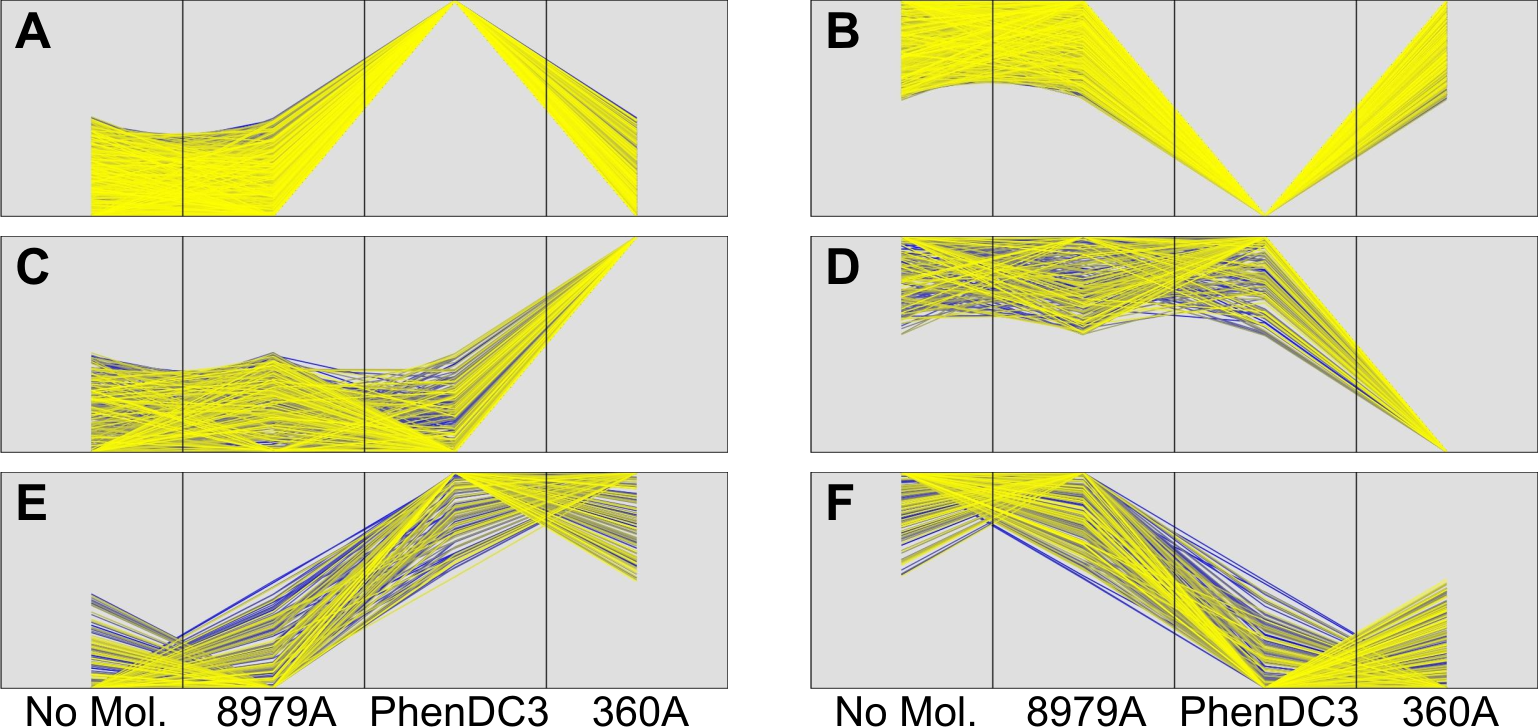
**Expression data plots for different categories: A) PhenDC3 up-regulated, B) PhenDC3 down-regulated, C) 360A up-regulated, D) 360A down-regulated, E) up-regulated with both compounds, F) down-regulated with both compounds**.

Whole genome expression of genes was determined using Illumina HumanHT-12v4 expression BeadChips after 48 hours of treatment with 10 μM of the G-quadruplex-specific molecules PhenDC3 or 360A in HeLa S3 cells. To account for non-specific effects mediated by the bisquinolinium compounds, 8979A (Figure 1C) was used as a control as it has a similar structure and charges but displays a very limited binding affinity to G-quadruplex motifs [43]. All molecule treatments were performed in triplicate. For analysis, the intensity values of each sample were subjected to 'quantile normalization'. The expression data was analyzed using the CLANS software [48]. First, the two control sets (untreated and 8979A-treated) samples were compared to confirm the non-specificity of the control molecule. Our analysis identified only 85 and 117 illumina identifiers down- and up-regulated, respectively, under the control condition. Next, we compared the expression of PhenDC3- or 360A-treated samples with the control samples (untreated and 8979A treated) to exclude those genes which might have been affected in their expression by treatment with the control molecule. We therefore considered the untreated and the 8979A-treated sample each as a control. Only genes showing a correlation coefficient of ≥ 0.9 to our conditions of interest (Figure 2) and an anova P-value ≤ 0.05 were taken into account and constitute our set of differentially expressed genes. The detailed gene list is given in Additional file 2: Table S4.

### G-quadruplex motifs in differentially expressed genes

Using the Genbank files from NCBI (build 36.2), we could map 52,980 G-quadruplex motifs within the 2 kb region up- and downstream of the TSS of 17,384 mRNAs (see methods for details). For further analysis we considered only those mRNAs for which the Illumina IDs are present in the differentially expressed set, which gave a final set of 1,157 down- and 1,529 up-regulated Illumina IDs for PhenDC3 and 249 down- and 401 up-regulated for 360A, respectively (Table 1). We found that, in terms of differentially expressed genes, 81.5% (1,804 out of 2,212) of PhenDC3-treated and 70% (369 out of 526) of 360A-treated genes have at least one PG4 motif within the ± 2 kb region of the TSS, which is significantly higher (hypergeometric test; 7.13E^-98 ^(PhenDC3) and 0.01 (360A)) than the whole genome average (67.3%; 16,607 out of 24,651). This indicated a specificity and noticeable effect on transcription of both bisquinolinium compounds towards G-quadruplex motifs under our experimental conditions. We also found that 185 and 287 genes were up- or down-regulated in both bisquinolinium-treated samples, respectively (Figure 2E-F).

**Table 1:** The distribution of G4 motifs in differentially expressed genes (Illumina IDs) after PhenDC3 and 360A treatment

	Illumina IDs	No. of Genes (with G4 motif)	No. of G4 motifs	Average loop length
unchanged^a^			9,456	3.7

8979A -up-regulated^b^	117	83		

8979A -down-regulated^b^	85	55		

PhenDC3 - up-regulated^b^	1529	1242 (975)	3,566	3.7

PhenDC3 - down-regulated^b^	1157	970 (829)	2,405	3.7

360A - up-regulated^b^	401	312 (233)	772	3.8

360A - down-regulated^b^	249	215 (136)	337	3.8

^a ^non-differentially expressed genes common in all the gene sets, anova > 0.75

^b^differentially expressed genes, anova ≤ 0.5 and correlation ≥ 0.9

**Figure 3:**
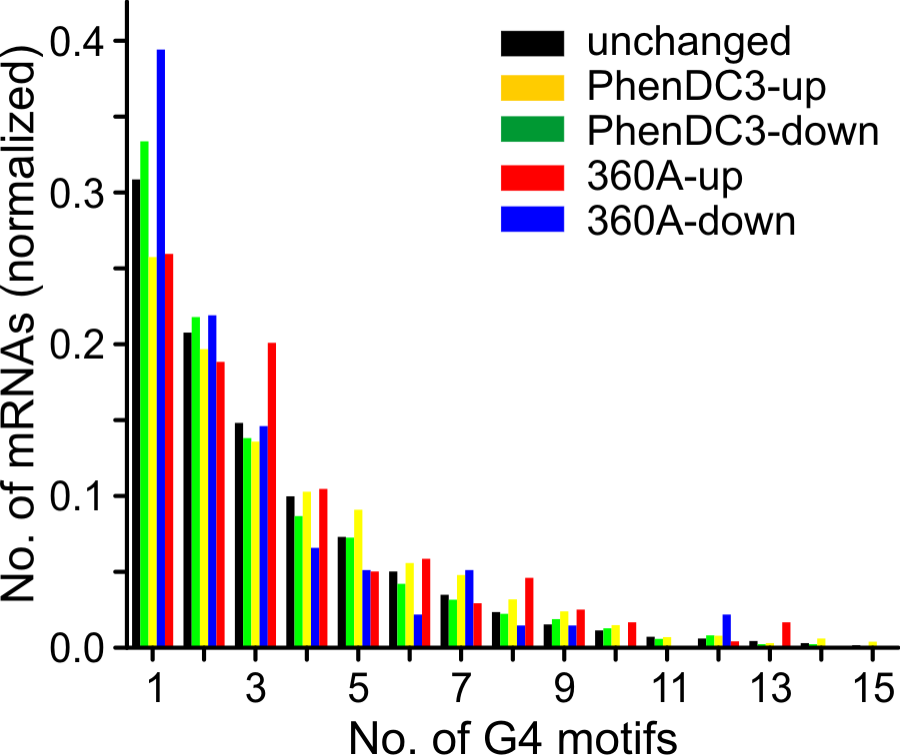
**Distribution of the number of G-quadruplex motifs in the core promoter region of differentially expressed and unchanged genes**. Data with more than 16 G-quadruplex motifs in the promoter are not shown (less than 1% of total)

Next we analyzed whether we could detect any difference in the distribution of G-quadruplex motifs in our up- and down-regulated set of genes. Surprisingly, the normalized distribution of down-regulated mRNAs shows a higher occurrence of one or two G-quadruplex motifs in their core promoter region (± 2 kb of TSS) compared to unchanged or up-regulated mRNAs. In contrast to this, genes with four or more G-quadruplexes are over-represented in the set of up-regulated mRNAs (Figure 3). To further validate the microarray results, we randomly chose 8 genes for quantitative real time PCR: Four genes showed changes in expression under treatment with both molecules (*CTPS2, DPY30, DIO2, EGFR*) and 2 genes each showed changes with either one of the molecules (PhenDC3: *CANX *and *AK1*; 360A: *PFKM *and *SH3GL1*). To further check whether the changes in gene expression show a dependency with respect to compound concentrations the cells were treated with 4 different concentrations (10, 20, 50 and 100 μM) of the G-quadruplex specific molecules PhenDC3 and 360A for 48 hours. We also used minimum (10 μM) and maximum (100 μM) concentrations of the control bisquinolinium 8979A. In addition, expression of *TGOLN2 *was also analyzed as a control because it did not appear to induce significant changes in expression in the microarray experiments. As a result of the real-time PCR data we were able to ascertain that all genes exhibited changes in their mRNA levels with a trend consistent to the microarray results (see Table 2). Except *CANX*, all genes showed a concentration-dependent increase or decrease in expression.

**Table 2:** Quantitative Real Time PCR in HeLa S3 cells after treating with increasing concentrations of different molecules.

	CTPS2	DPY30	DIO2	EGFR	CANX	AK1	PFKM	SH3GL1	TGOLN2
8979A-10 uM	1.18 (▬)	-1.23 (▬)	1.13 (▬)	-1.79 (▬)	-1.45 (▬)	2.02 (▬)	-1.99 (▬)	1.61 (▬)	-1.29 (▬)

8979A-100 uM	1.28 (▬)	1.00 (▬)	-1.37 (▬)	1.95 (▬)	1.14 (▬)	-1.91 (▬)	1.06 (▬)	1.95 (▬)	1.82 (▬)

PhenDC3-10 uM	3.06 (▲)	-1.79 (▼)	1.35 (▲)	-1.39 (▼)	12.69 (▲)	-1.61 (▼)			1.06 (▬)

PhenDC3-20 uM	2.09 (▲)	-1.83 (▼)	1.41 (▲)	-1.65 (▼)	10.78 (▲)	-1.41 (▼)			

PhenDC3-50 uM	1.93 (▲)	-3.59 (▼)	1.99 (▲)	-1.78 (▼)	7.06 (▲)	-1.59 (▼)			

PhenDC3-100 uM	1.50 (▲)	-3.60 (▼)	2.19 (▲)	-1.99 (▼)	6.42 (▲)	-1.76 (▼)			1.04 (▬)

360A-10 uM	3.01 (▲)	-2.06 (▼)	-1.24 (▼)	2.41 (▲)			1.86 (▲)	1.29 (▲)	1.43 (▬)

360A-20 uM	1.65 (▲)	-1.11 (▼)	-1.08 (▼)	2.42 (▲)			4.13 (▲)	3.73 (▲)	

360A-50 uM	3.73 (▲)	-1.89 (▼)	-1.69 (▼)	6.23 (▲)			1.87 (▲)	1.25 (▲)	

360A-100 uM	8.22 (▲)	-1.22 (▼)	-2.17 (▼)	13.11 (▲)			2.23 (▲)	2.34 (▲)	-1.12 (▬)

The status in microarray is shown in parenthesis as unchanged (▬), upregulated (▲) or downregulated (▼)

### Effect of position and strand occupancy of G-quadruplex motifs

**Figure 4:**
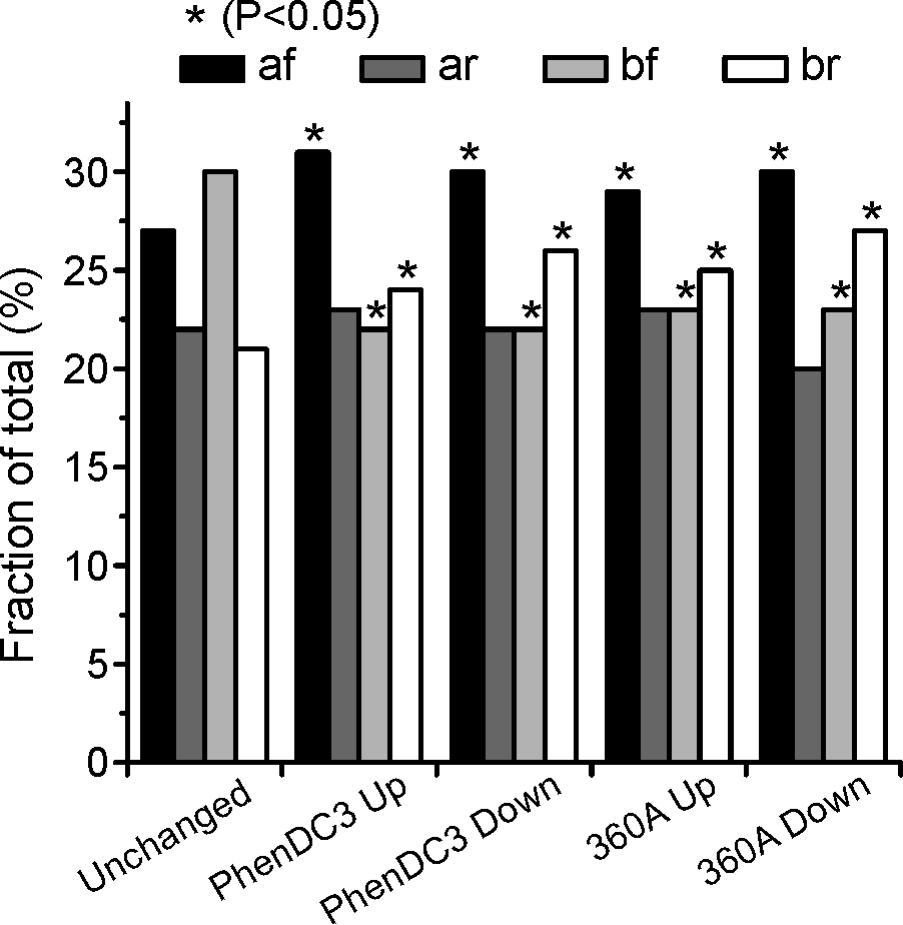
**Distribution of G-quadruplex motifs with respect to TSS (before or after) and DNA strand (forward or reverse) in af(after-forward), ar(after-reverse), bf (before-forward) and br (before-reverse) subsets**.

To investigate the effect of the position of G-quadruplexes with respect to the TSS and strand orientation, we further compared the number of G-quadruplexes in the up- and down-regulated genes with the set of genes showing no significant change in expression that also harbored quadruplexes in their ± 2 kb region (non-differentially expressed genes common between all the gene sets, anova > 0.75). These were divided into 4 subsets each: 1) **af**: G4-motifs present after TSS on forward strand, 2) **ar**: after TSS on reverse strand, 3) **bf**: before TSS on forward strand and 4) **br**: before TSS on reverse strand (Additional file 1: Table S1). Interestingly, we found that the number of G-quadruplexes in category **af **and **br **are significantly higher for the up- and down-regulated genes (both examined molecules) than unchanged/non-differentially expressed genes and have lower representation for category **bf **(hypergeometric test, p < 0.05, Figure 4).

### G-quadruplex motif loop length and composition in differentially expressed genes

In order to search for a consensus motif or common feature that might be responsible for the differing behavior in non-affected and differentially expressed genes in response to the compound treatment, we investigated the difference in sequence composition between the G-quadruplex motifs present in differentially expressed genes versus genes with unchanged expression. The loop length and its nucleotide composition has been extensively studied earlier and both are known to affect the thermodynamic stability of G-quadruplex motifs [49-51]. Hence, we first examined the average loop length of G-quadruplex motifs occurring in differentially expressed versus non-differentially expressed genes. As shown in Table 1 the average loop lengths of quadruplexes in the different sets turned out to be very similar.

**Figure 5:**
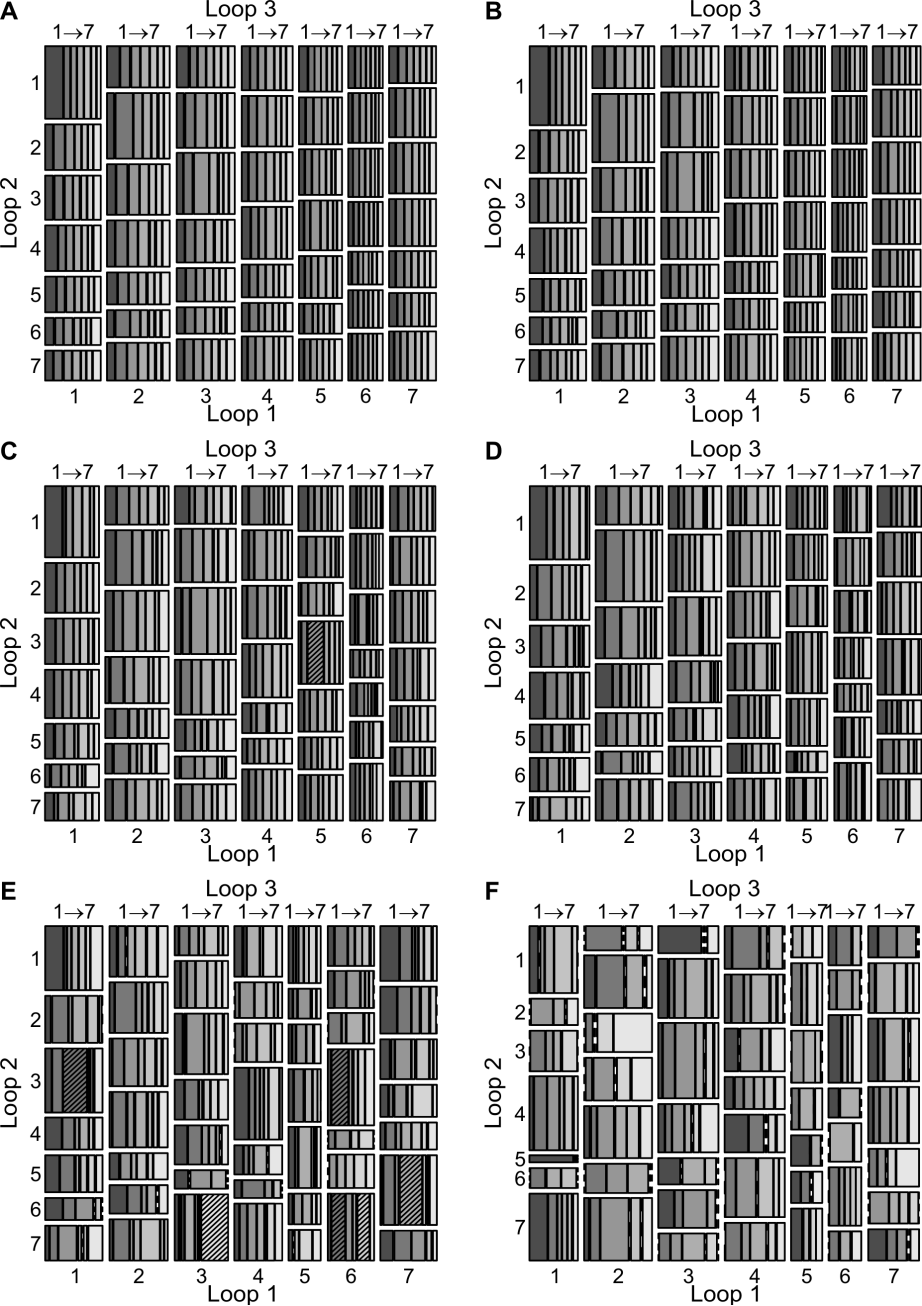
**Mosaic plot representation of loop length distribution of PG4 motifs present in ± 2 kb surrounding the TSS in A) whole genome (± 2 kb of TSS, n = 51,793), B) Unchanged gene set (n = 17,580) C) PhenDC3 upregulated (n = 3,471), D) PhenDC3 down-regulated (n = 2,354), E) 360A up-regulated (n = 751) and F) 360A down-regulated (n = 328)**. Significantly different loop combinations are highlighted with a hatching pattern. Individual loop combinations with no data are drawn with a dotted line.

We next looked further into the individual loop lengths of the three loops (loop 1, 2 and 3 numbered 5' to 3' end) of the G-quadruplex motif. The G-quadruplex motifs with more than four GGG repeats are not considered for this analysis due to the uncertainty regarding which of the actual GGG repeats contribute to the 'stem' of the G-quadruplex motif. However, such sequences represented less than 2% of all G-quadruplex motifs. Interestingly, G-quadruplex motifs in the up-regulated genes exhibited significant changes in some loop length combinations (hypergeometric test, PhenDC3: [5-4-3], P = 3.27E^-06 ^and 360A: [1-3-3], P = 5.17E^-06^; [3-7-7], P = 5.52E^-08^; [6-4-2], P = 6.71E^-07^; [6-7-2], P = 2.91E^-04^; [6-7-6], P = 3.39E^-04^; [7-6-4], P = 3.31E^-08^; wherein [n-n-n] is [loop1-loop2-loop3], Figure 5C and 5E, respectively) compared to the mosaic plot of whole genome G-quadruplex loop length compositions and the quadruplexes found in the set of non-differentially expressed genes (Figure 5A and 5B). Although we could find some of the loop combinations significantly enriched using the hypergeometric test, these results could be partially affected due to the low number of G-quadruplexes in the 360A up-regulated gene set. Interestingly, the G-quadruplexes belonging to genes with unchanged expression did not show any significant changes in loop composition (Figure 5B). Noticeably, only longer loop length combinations exhibited higher representation in the PhenDC3 and 360A-up-regulated genes. One possible explanation might be that longer loops provide more surface for recognition by interaction partners as also concluded recently [52]. In the presence of G4-binding small molecules these interactions may be hindered leading to differential expression of the corresponding genes. The PhenDC3-down-regulated genes did not show any significant changes (Figure 5D), while the number of G-quadruplexes in 360A-down-regulated genes (n = 328) are less than the possible loop combinations (7 × 7 × 7 = 343) and hence this set was not tested for significance (Figure 5F).

**Figure 6:**
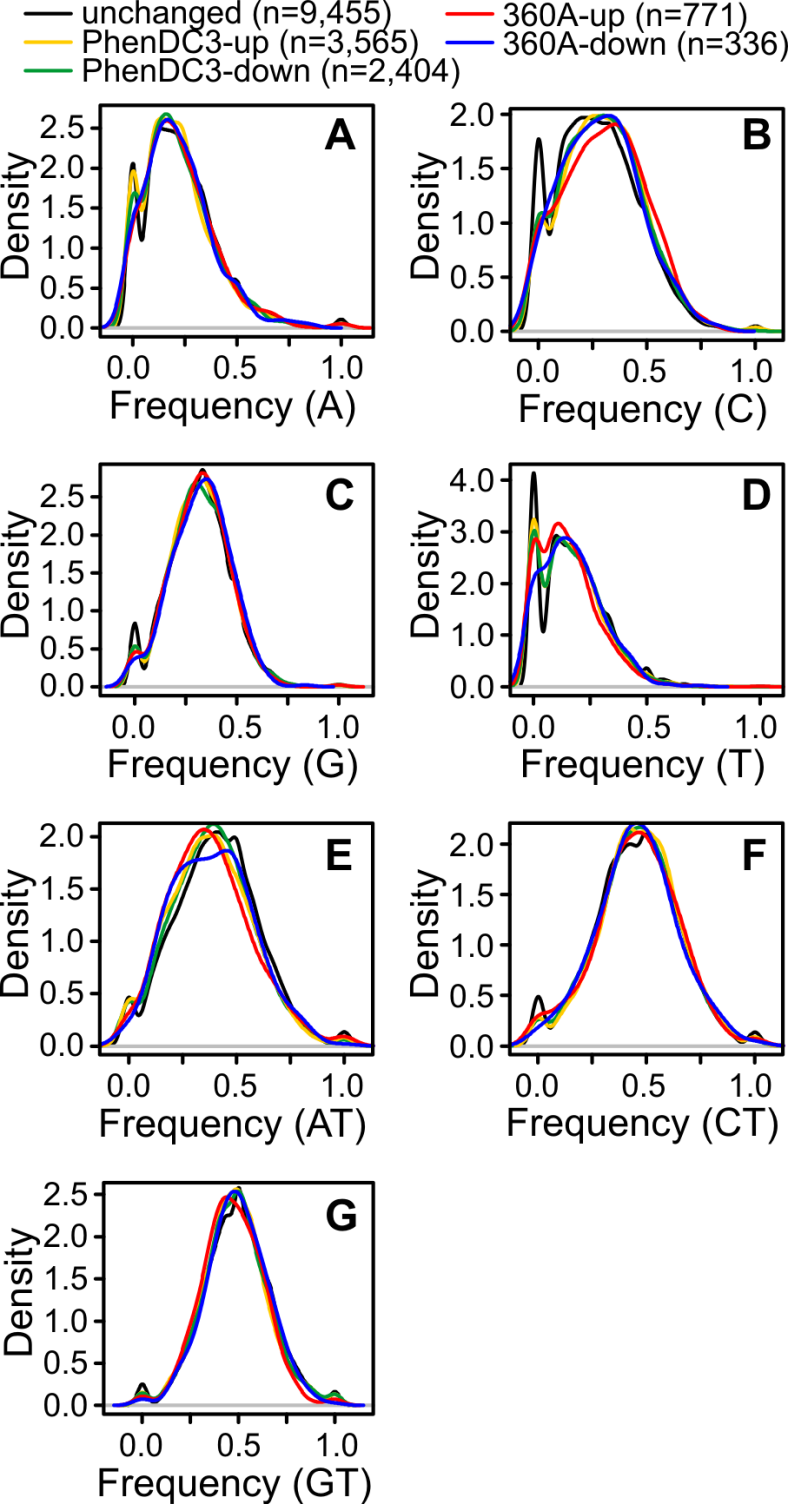
**Loop composition analysis of G-quadruplex motifs present in differentially expressed genes**. Kernel density plot showing the distribution of individual nucleotides (A) A, (B) C, (C) G and (D) T or nucleotide groupings (E) AT, (F) CT and (G) GT that occur in loops of G-quadruplex motifs. The number of G-quadruplex motifs used to plot the kernel density is shown in the parentheses.

We further examined the nucleotide composition in the loops of the G-quadruplex motifs in the differentially expressed and the unchanged genes. We checked for the frequency of individual nucleotides (A, C, G, and T) as well as groupings (AT, CT and GT) with respect to the total length of the three loops. The grouping AT represents the total number of A and T in the loops. The same is true for other groupings. The inverse of these groupings would be CG, AG and AC frequencies, respectively, which, combined, allows us to represent all possible nucleotide combinations. The kernel density plot [53], which is a non-parametric method of estimation of the frequency distribution of a finite and random variable, in present case the nucleotide(s) in the loops, were prepared using 'R' for all the individual nucleotides or groupings in different sets and compared with the reference (non-differential) set (described above). The frequency value can vary from 0 to 1, where '0' represents no loop nucleotide matching with the query nucleotide base(s) and '1' represents that all the nucleotides in the loop region are the same as the query nucleotide base(s). Incidentally, except for nucleotide 'C' the frequencies for all other nucleotides or nucleotide groupings in the loop regions were found to have similar distributions (Figure 6). In case of 'C' occurring in the loop region, G-quadruplexes in the unchanged expression gene set showed a higher incidence of complete exclusion of C (zero frequency) compared to the differentially expressed genes (Kolmogorov-Smirnov test, PhenDC3 up: P = 1.197E^-09^, PhenDC3 down: P = 1.407E^-04^, 360A up: P = 1812E^-05^, 360A down: P = 0.497). To further check if the position with respect to TSS or strand has any relevance between the up- and down-regulated genes, we analyzed the kernel density plots for subgroups **af**, **ar**, **bf**, and **br **(described above) for the differentially expressed genes. However, under these conditions, we could not find any significant changes in the loop distribution of the individual nucleotides or nucleotide groupings (Additional file 1: Figure S1). Taken together, no strong differences of loop compositions were detectable between the sets of genes that showed differential expression after compound treatment and the unchanged genes. However, the binding mode of the utilized bisquinolinium compounds is not known for the many different variations of quadruplexes targeted here. The exact binding mode has probably a large influence on how sequence variations will affect the changes of gene expression observed after compound treatment.

### Functional classification of the differentially expressed genes

To understand the biological relevance of the differentially expressed genes, we performed functional class scoring using Gene Ontology (GO) [54]. The differentially expressed genes after treatment with PhenDC3 exhibited enrichment (P < 0.01, after Bonferroni correction) in gene classes involved in various metabolic processes (P = 1.19E^-10^), gene expression (P = 2.15E^-07^), and regulation of transcription by RNA pol II (P = 0.0003). Similar enrichment of metabolic pathways (transcription regulation by RNA pol II) was noticed earlier [53]. On the other hand, genes belonging to the immune response (P = 0.0002) and G-protein-coupled signal transduction (P = 0.006) were significantly depleted (Table 3). The first result is likely caused by the fact that most genes in this class are silent in the examined cell line. In case of 360A treated cells no GO class was significantly enriched or depleted after correction for multiple testing, however, without Bonferroni correction, enrichment in classes for cell cycle, protein transport, and localization was found (Additional file 1: Table S3) which was also shown for TMPyP4-treated cells before [55]. The enrichment of the differentially expressed genes in various metabolic processes and protein transport pathways could pose a challenge for estimating the therapeutic consequences of G-quadruplex specific molecules [19,56] which are thought to act at least in part via suppression of transcription of individual oncogenes [20,25,28,57].

**Table 3:** Gene Ontology (GO) categories enriched/depleted (P < 0.01) of differentially expressed genes after treating HeLa S3 cells with PhenDC3

GO ID	TERM	P VALUE
**Enriched GO classes**		

GO:0044237	cellular metabolic process	2.42E-12

GO:0034960	cellular biopolymer metabolic process	5.07E-12

GO:0044260	cellular macromolecule metabolic process	2.90E-11

GO:0043283	biopolymer metabolic process	5.91E-11

GO:0008152	metabolic process	1.19E-10

GO:0043170	macromolecule metabolic process	2.94E-10

GO:0044238	primary metabolic process	8.10E-10

GO:0006139	nucleobase, nucleoside, nucleotide and nucleic acid metabolic process	1.91E-07

GO:0010467	gene expression	2.15E-07

GO:0009987	cellular process	4.16E-07

GO:0043284	biopolymer biosynthetic process	1.28E-06

GO:0034961	cellular biopolymer biosynthetic process	1.28E-06

GO:0016070	RNA metabolic process	1.90E-06

GO:0034645	cellular macromolecule biosynthetic process	5.34E-06

GO:0009059	macromolecule biosynthetic process	8.73E-06

GO:0046907	intracellular transport	0.0001430

GO:0051641	cellular localization	0.0002268

GO:0006357	regulation of transcription from RNA polymerase II promoter	0.0003508

GO:0044249	cellular biosynthetic process	0.0007565

GO:0009058	biosynthetic process	0.0009248

GO:0051649	establishment of localization in cell	0.0013971

GO:0044267	cellular protein metabolic process	0.0075149

**Depleted GO classes**		

GO:0002376	immune system process	0.0074009

GO:0007186	G-protein coupled receptor protein signaling pathway	0.0063202

GO:0007166	cell surface receptor linked signal transduction	0.0002492

GO:0006955	immune response	0.0001782

GO:0032501	multicellular organismal process	4.93E-06

## Conclusions

In summary, the present study shows that the bisquinolinium compounds PhenDC3 and 360A can induce G-quadruplex-motif-specific changes in transcription. In contrast to previous studies, our experiments employ more quadruplex-specific compounds, which is reflected in the over-representation of differentially regulated genes bearing quadruplex motifs in their promoter regions. In an earlier study using TMPyP4 as a more non-specific G-quadruplex binder 61% of the differentially expressed genes were connected to the occurrence of a G4 motif in their ± 1 kb region around TSS which is less than expected [5]. In comparison, PhenDC3 and 360A showed enhanced specificity. The differences observed between the two compounds can result from slight variations in affinity for various quadruplex-forming sequences as shown recently [58]. However, we were unable to detect obvious characteristics such as loop compositions and motifs common to quadruplexes that are associated with genes that showed changes. Such identifiers would also have been very interesting to observe also with respect to the therapeutic potential of quadruplex-targeting small molecules. On the other hand, given the rather late time point of 48 h post incubation with the quadruplex-inducing compound, we cannot rule out that some or many of the observed effects are secondary and therefore cannot be attributed to a direct effect of a quadruplex formation. Taking this into account, future studies of quadruplex-targeting compounds should also take into account earlier time points in order to ensure that indeed primary changes of gene expression are observed.

Interestingly, we found that more genes were up-regulated than down-regulated following compound treatment. Several studies reported quadruplexes as transcription suppressor elements when their effects on individual genes were studied [19,28,59]. However, positive effects on transcription levels via the formation of promoter G-quadruplexes have also been described [60,61] and hence the influence of the formation of a promoter-based quadruplex on transcription is very likely dependent on the exact position and the promoter architecture. Similar up-regulation effects were noticed earlier in genome-wide microarray experiments following TMPyP4 [55] or single-chain antibody [38] treatments. Similarly, in absence of WRN/BLM helicases which are believed to resolve G-quadruplex motifs, a strong association was observed between up-regulated genes and G-quadruplex motifs in promoter regions [62]. It has been speculated that the changes could be mediated by an influence of the G4 motifs on the chromatin state [10,12]. It is also possible that the absence of a helicase (or in our case stabilization of transient G-quadruplex motifs by small molecules) may result in formation of transcription factor binding sites or destruction of repressor binding sites. In addition, along with other non-canonical structures such as cruciform and triplexes, quadruplexes have been discussed as modulators of superhelical torsion. Stress-induced duplex destabilization (SIDD) is more prevalent in transcriptional regulatory sites [63] and quadruplex formation was shown to release supercoiling stress [6]. The occurring denaturation of duplex DNA in regulatory regions near the transcription start site would be an easy way of interfering with the transcriptional machinery and its regulation. However, more detailed studies are required to further decipher the exact mechanisms underlying quadruplex-mediated changes in transcription. We also observed a positional bias in the distribution of G-quadruplex motifs in differentially expressed genes. G-quadruplexes are enriched on the reverse strand before the TSS or on the forward strand after the TSS when compared to the G4-motifs present in non-differentially expressed genes. Interestingly, G4 motifs present on the non-template strand have been shown to play a role in RNA pol II pausing [64], which provides a possible mechanism for how these may in turn regulate the expression of the downstream gene. Further, a similar distribution of the loop size and composition of G-quadruplexes between the perturbed and non-perturbed genes were found, suggesting the precise genomic position and the composition of the surrounding DNA are likely to play an important role in determining the effect a given quadruplex will have on gene-transcription.

## Methods

### RNA Isolation and microarray experiments

HeLa S3 cells (obtained from the DSMZ, the German Resource Center for Biological Materials, http://www.dsmz.de/) were cultured in DMEM high glucose medium with 10% Fetal Calf Serum (FCS). For PhenDC3, 360A and 8979A, 10 mM stock solutions were prepared in DMSO and diluted directly in the DMEM medium to 10 μM just before using. The HeLa S3 cells exhibited 92% and 100% cell viability at 10 μM PhenDC3 and 360A, respectively, after 48 hrs treatment. After 48 hours of treatment, the whole RNA was isolated using RNeasy plus mini kit (Qiagen). Microarray experiment was performed by Aros Applied Biotechnology (Denmark) as per the manufacturer's protocol. Briefly, RNA was amplified using Illumina TotalPrep RNA amplification kit and hybridized on Illumina HumanHT-12v4 expression BeadChips as recommended by the manufacturer. Hybridization data was obtained by iScan Bead Array scanner (Illumina) and preprocessed by quantile normalization (Compliance to MIAME guidelines).

### Microarray data analysis

The intensity values after quantile normalization for all triplicate experiments were further analyzed using CLANS software http://bioinfoserver.rsbs.anu.edu.au/downloads/clans/. Briefly, all the Illumina IDs (probes) were assumed to be differentially expressed if their anova values were ≤ 0.5 and to be of interest if their correlation to a condition of interest (see above, table or figure listing the 8 conditions) was ≥ 0.9. All experiments were performed in triplicate and overall > 98% correlation was observed between replicates.

### Data retrieval and G-quadruplex mapping

The Genbank (.gbk)files for the Homo sapiens reference genome build 36.2 were downloaded from the NCBI ftp site ftp://ftp.ncbi.nih.gov/genomes/H_sapiens/ARCHIVE/BUILD.36.2. The G-quadruplex motifs were identified using a custom PERL program. The motif searched for was 5'-(GGGN_1-7_)_≥3_GGG-3', wherein N = A, T, G or C. All G-quadruplex motifs were mapped to their nearest mRNA within ± 2 kb of Transcription Start Sites (TSS). To be certain the same sequence build was used for all analyses, we independently mapped the Illumina probe sequences (47,323) onto the available mRNA data using BLAT [65] and also the mRNA sequences (45,495; retrieved from the Genbank files) onto the Illumina IDs. One Illumina ID may map to multiple mRNAs and vice versa, due to the non-equivalence of the datasets. This resulted in 32,980 mRNAs mapping to 34,638 Illumina probe IDs. The 32,980 mRNAs could be matched to 24,651 NCBI genes. Next, we also mapped the G-quadruplexes present (53,397) in the ± 2 kb region of the TSS of the 17,384 mRNAs (16,607 genes). The terms "forward", "reverse", "before TSS", and "after TSS" used to classify quadruplexes refer to the position the G-rich quadruplex is found with respect to the coding (sense or template) strand of the associated gene. In this respect "forward" means the presence of a quadruplex on the coding (sense or template) strand.

### Real time polymerase chain reaction

Two step real time PCR was performed to validate the microarray results. cDNA was prepared by using M-MuLV reverse transcriptase (Fermentas). Relative quantitative real time PCR was performed using LightCycler 480 SYBR Green I master (Roche) in a Roche LightCycler 480 system, for primer sequences see additional file 1, Figure S2. A negative control for each gene with no template was included. Reaction conditions consisted of 95°C for 1 min followed by 45 cycles of amplification (15 sec at 95°C, 15 sec at 60°C and 15 sec at 72°C). After amplification, melting analysis was performed to ensure a uniform amplification product.

### Loop length and composition analysis

The different features (length and composition) of the G-quadruplex motifs were extracted via a PERL script and visualized using 'R' http://www.r-project.org/. For programmatic simplicity, the first three guanines from 5'-end were considered part of the stem and consecutive guanines, if any, were considered as part of the loop (length 1-7). To reduce redundancy in loop length and composition, each G-quadruplex motif was considered only once. All the statistical tests were performed in 'R' http://www.r-project.org/.

### Gene Ontology (GO) classification

For functional classification all the differentially expressed gene names were converted to Uniprot standard gene name using gene merge (http://genemerge.cbcb.umd.edu/GeneMerge.php, [66]) and these converted gene names were used to obtain the GO category for biological function (http://genome.crg.es/GOToolBox/, [54]). Bonferroni correction was used to correct for multiple testing for PhenDC3. NOTE: The results for the 360A treated sample were not corrected for multiple testing as this removed all significant hits. These results should be interpreted with this in mind. Categories having p-value less than 0.01 was considered to be significantly enriched/depleted.

### Availability of supplementary data

Further data sets including are available as Additional file 1 (containing Figure S1 and Tables S1, S2, and S3) and Additional File 2 (Table S4). The microarray data set supporting the results of this article is available online at the Gene Expression Omnibus repository (accession number GSE32170, http://www.ncbi.nlm.nih.gov/geo/query/acc.cgi?token=rnaxlqiuomkuips&acc=GSE3217)

## Competing interests

The authors declare that they have no competing interests.

## Authors' contributions

Conceived and designed the experiments: All authors. Performed the experiments: RH. Analyzed the data: RH, TF, JSH. Contributed reagents: RH JFR MPTF TF. Wrote the manuscript: RH, TF, JSH. All authors read and approved the final manuscript.

## Supplementary Material

Additional file 1 **Table S1**. Number of G-quadruplexes present in different subsets. **Table S2**. Primers used for quantitative real time PCR. **Table S3**. Enriched Gene Ontology (GO) classes of differentially expressed genes after treating HeLa S3 cells with 360A. **Figure S1**. Kernel density plot showing the distribution of individual nucleotides (A) A, (B) C, (C) G and (D) T or nucleotide groupings (E) AT, (F) CT and (G) GT that occur in loops of G-quadruplex motifs in af, ar, bf and br subsets (see Table S2). Click here for file

Additional file 2 **Table S4 in excel-format: List of all differentially expressed genes**. Click here for file
