# Supplementary material for: Bisquinolinium compounds induce quadruplex-specific transcriptome changes in HeLa S3 cell lines
Source: BMC Res Notes. 2012 Mar 13;5:138. doi: 10.1186/1756-0500-5-138 (PMC3375199; doi:10.1186/1756-0500-5-138)

## Supporting Information for:

## Bisquinolinium Compounds induce Quadruplex-Specific

## Transcriptome Changes in HeLa S3 Cell Lines.

Rashi Halder,^1^ Jean-Francois Riou,^2^ Marie-Paule Teulade-Fichou,^3^ Tancred Frickey,^4^ and Jörg S. Hartig^1*^

1: Department of Chemistry and Konstanz Research School Chemical Biology (KoRS-CB), University of Konstanz, Universitätsstraße 10, 78457 Konstanz, Germany

2: Structure des Acides Nucléiques, Télomères et Evolution, INSERM U565, CNRS UMR 7196, Muséum National d’Histoire Naturelle, 43 rue Cuvier, 75231 Paris cedex 05, France

3: Institut Curie, UMR 176-CNRS, Bât 110, Université Paris-Sud, 91405 Orsay, France

4: Department of Biology, University of Konstanz, Universitätsstraße 10, 78457 Konstanz, Germany

*To whom correspondence should be addressed. Tel: +49 7531 882398; Fax: +49 7531 885140; Email: [joerg.hartig@uni-konstanz.de](mailto:joerg.hartig@uni-konstanz.de)

Table S2 2

Table S3 3

Table S4 4

Figure S1 5

**Table S2**. Number of G-quadruplexes present in different subsets.

|  | bf | br | af | ar |
| --- | --- | --- | --- | --- |
| PhenDC3 and 360A – unchanged | 3311 | 2294 | 2998 | 2419 |
| PhenDC3 – upregulated | 840 | 907 | 1202 | 889 |
| PhenDC3 – downregulated | 592 | 707 | 810 | 589 |
| 360A – upregulated | 189 | 207 | 238 | 187 |
| 360A – downregulated | 85 | 100 | 111 | 74 |

bf: before forward, br: before reverse, af: after forward, ar: after reverse


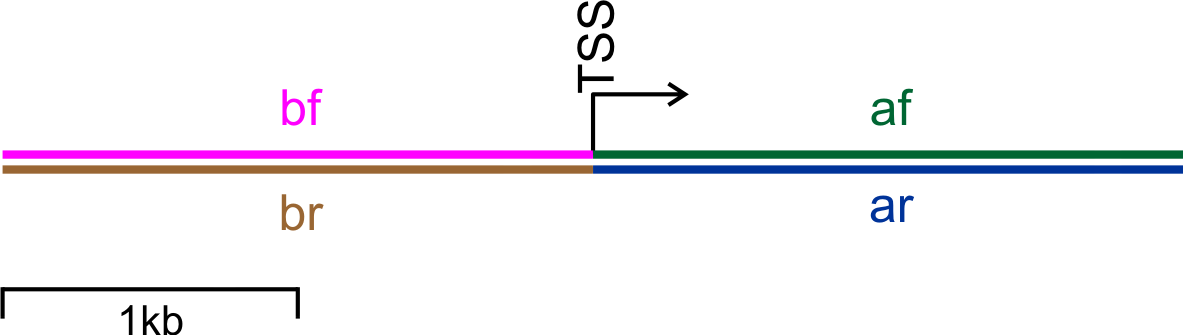


**Table S3**. Primers used for quantitative real time PCR

| Gene symbol | Forward primer (5’-3’) | Reverse primer (5’-3’) |
| --- | --- | --- |
| 18S | GTAACCCGTTGAACCCCATT | CCATCCAATCGGTAGTAGCG |
| B2M | TGCTGTCTCCATGTTTGATGTATCT | TCTCTGCTCCCCACCTCTAA |
| CTPS2 | CCCAGTCTCATTGTTCCTCC | CCACAGAGTTTAGGCCAAATG |
| DPY30 | CGAGTTGGCAAAGACTGGAG | GAGTACGGTCTCACAGACAACG |
| DIO2 | TTGCTAAAACCTGATGGAGGA | CATGCTGACCTCAGAGGGA |
| EGFR | GTGACCGTTTGGGAGTTGATGA | GGCTGAGGGAGGCGTTCTC |
| CANX | GATGACTGGGATGAAGATGC | TCACATCTAGGGTTGGCAAT |
| PFKM | CCCTGACAGCAGCATTCATA | ATCATGACCCATGAAGAGCA |
| SH3GL1 | GCTGGTGACATCCACCTTCT | AGTTCTACAAGGCGAGCCAG |
| AK1 | TGTGAGAAGATCGTGCAGAA | CATGTCCAACACTGTCTCCA |
| TOGLN2 | TCAAAGCTGAAACGAGAGCA | AAGGCCAGTGACTACCAACG |

**Table S4.** Enriched Gene Ontology (GO) classes of differentially expressed genes after treating HeLa S3 cells with 360A

| **GO ID** | **TERM** | **P VALUE** |
| --- | --- | --- |
| GO:0051234 | establishment of localization | 0.0002727 |
| GO:0006810 | transport | 0.0003710 |
| GO:0007049 | cell cycle | 0.0004002 |
| GO:0016192 | vesicle-mediated transport | 0.0005539 |
| GO:0051641 | cellular localization | 0.0006009 |
| GO:0007041 | lysosomal transport | 0.0007979 |
| GO:0051649 | establishment of localization in cell | 0.0008467 |
| GO:0046907 | intracellular transport | 0.0009386 |
| GO:0007034 | vacuolar transport | 0.0018062 |
| GO:0033036 | macromolecule localization | 0.0018339 |
| GO:0051179 | localization | 0.0019069 |
| GO:0009411 | response to UV | 0.0023047 |
| GO:0008104 | protein localization | 0.0023879 |
| GO:0006897 | endocytosis | 0.0026701 |
| GO:0010324 | membrane invagination | 0.0026701 |
| GO:0016044 | membrane organization | 0.0029434 |
| GO:0006613 | cotranslational protein targeting to membrane | 0.0039452 |
| GO:0006622 | protein targeting to lysosome | 0.0039452 |
| GO:0015031 | protein transport | 0.0043308 |
| GO:0016197 | endosome transport | 0.0043486 |
| GO:0008543 | fibroblast growth factor receptor signaling pathway | 0.0045231 |
| GO:0045184 | establishment of protein localization | 0.0048901 |
| GO:0015992 | proton transport | 0.0058074 |
| GO:0007242 | intracellular signaling cascade | 0.0058668 |
| GO:0034613 | cellular protein localization | 0.0067155 |
| GO:0070727 | cellular macromolecule localization | 0.0069307 |
| GO:0022402 | cell cycle process | 0.0071530 |
| GO:0051246 | regulation of protein metabolic process | 0.0072770 |
| GO:0006818 | hydrogen transport | 0.0072990 |
| GO:0006886 | intracellular protein transport | 0.0073403 |
| GO:0009987 | cellular process | 0.0077087 |
| GO:0006605 | protein targeting | 0.0081032 |
| GO:0030518 | steroid hormone receptor signaling pathway | 0.0086020 |
| GO:0006766 | vitamin metabolic process | 0.0086020 |
| GO:0009314 | response to radiation | 0.0090504 |
| GO:0009755 | hormone-mediated signaling | 0.0099408 |
| GO:0032886 | regulation of microtubule-based process | 0.0099561 |

**Figure S1.** Kernel density plot showing the distribution of individual nucleotides (A) A, (B) C, (C) G and (D) T or nucleotide groupings (E) AT, (F) CT and (G) GT that occur in loops of G-quadruplex motifs in af, ar, bf and br subsets (see Table S2).


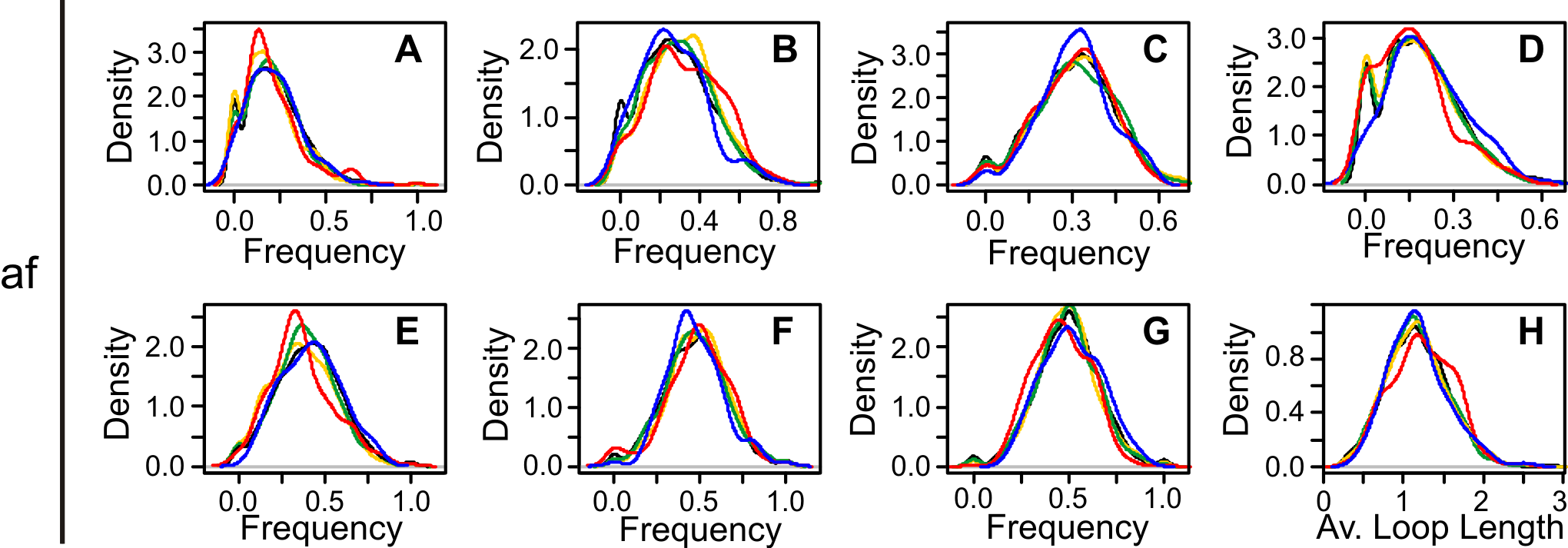


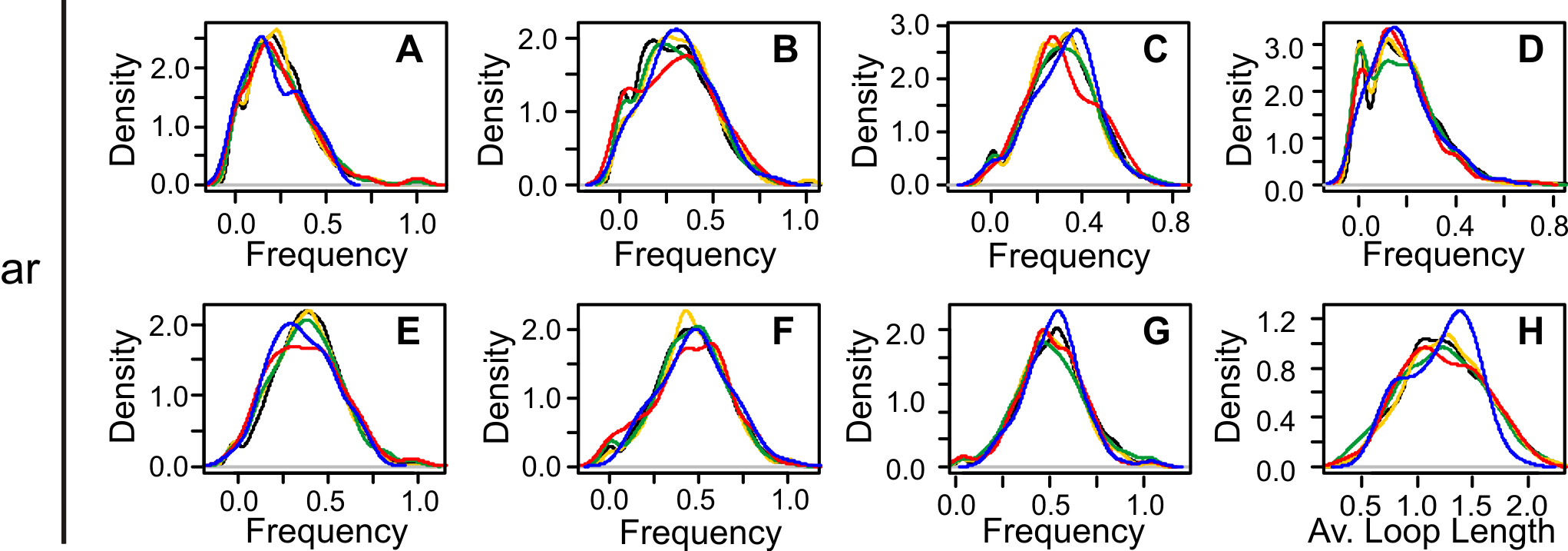


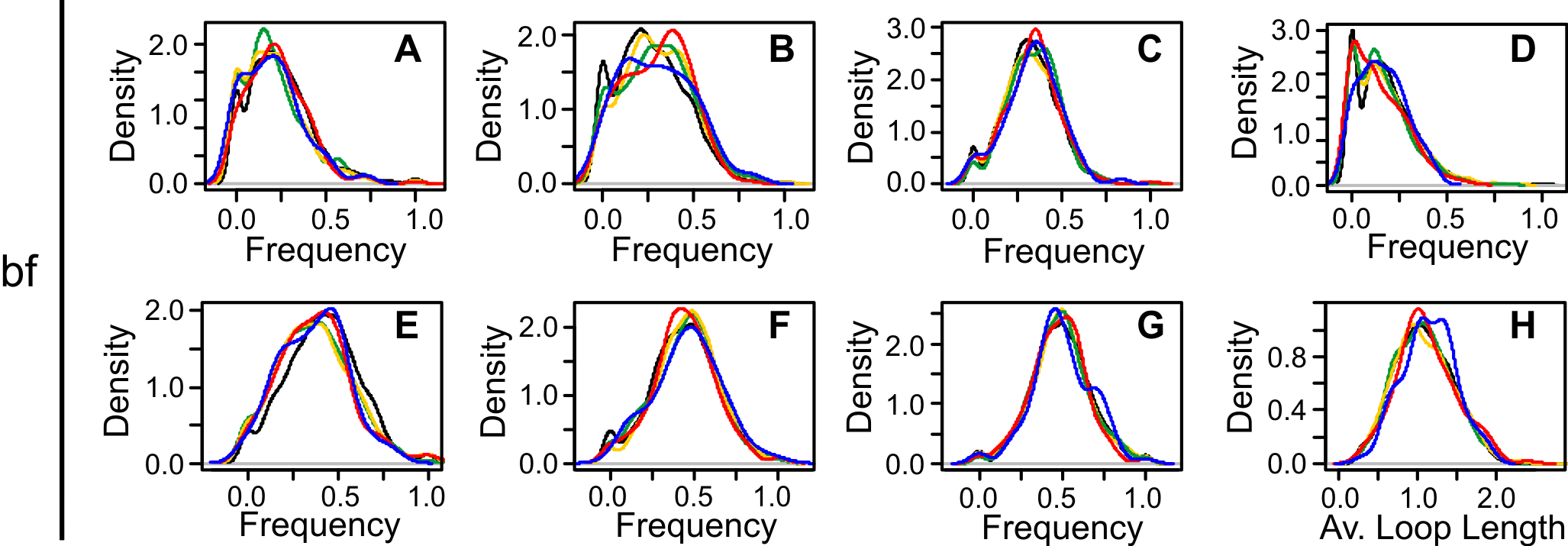


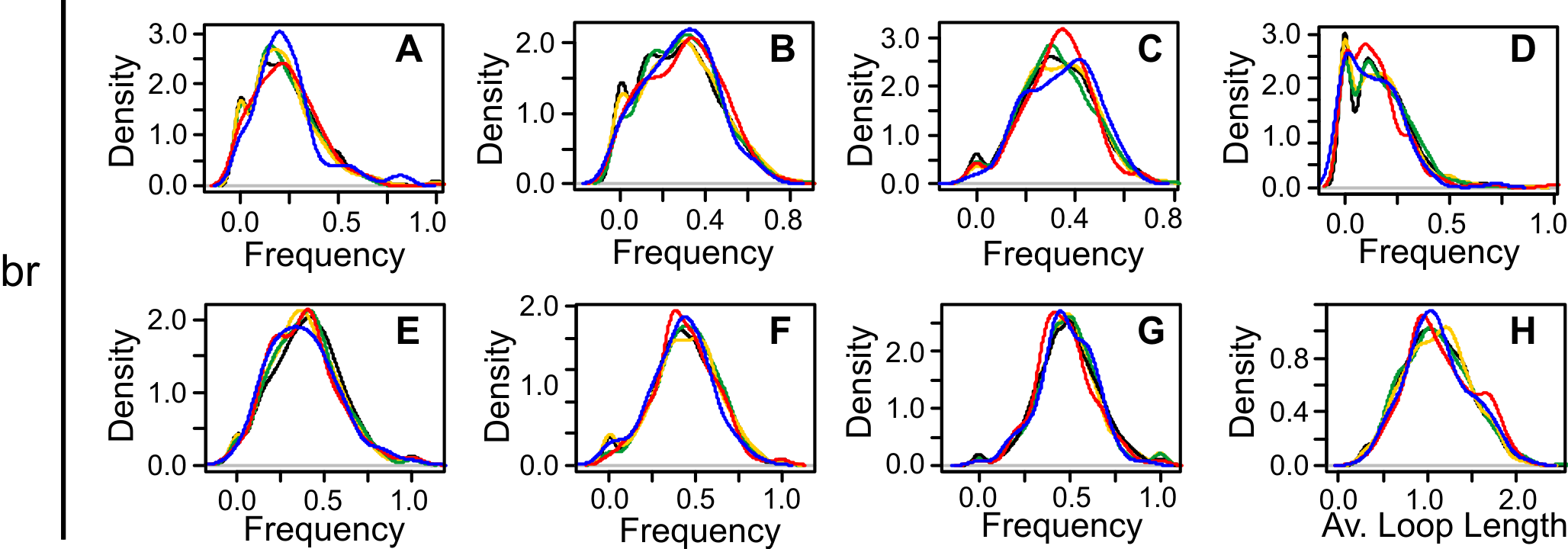

Supplement: Additional file 1 — Table S1. Number of G-quadruplexes present in different subsets. Table S2. Primers used for quantitative real time PCR. Table S3. Enriched Gene Ontology (GO) classes of differentially expressed genes after treating HeLa S3 cells with 360A. Figure S1. Kernel density plot showing the distribution of individual nucleotides (A) A, (B) C, (C) G and (D) T or nucleotide groupings (E) AT, (F) CT and (G) GT that occur in loops of G-quadruplex motifs in af, ar, bf and br subsets (see Table S2). [file 1756-0500-5-138-S1.DOC]
